# Simulation analysis of the preventative effects of planting sweet corn on nitrate leaching in a cherry greenhouse soil

**DOI:** 10.3389/fpls.2024.1482292

**Published:** 2024-11-01

**Authors:** Sen Hou, Quanjuan Fu, Huifeng Li, Rui Gao, Yugang Sun, Guoqin Wei

**Affiliations:** ^1^ National Laboratory Institution, State Key Laboratory of Nutrient Use and Management, Jinan, Shandong, China; ^2^ Shandong Institution of Pomology, Taian, Shandong, China

**Keywords:** greenhouse, catch crop, sweet corn, residual nitrate, nitrate leaching, water drainage, HYDRUS-1D model

## Abstract

**Introduction:**

To ensure higher productivity, fertilizers have been excessively applied to the fruit greenhouse soil yearly, thus resulting in the increasing risks of residual nitrate leaching in the North China Plain.

**Methods:**

In this study, a water and solute transport HYDRUS-1D model was used to evaluate the effects of using sweet corn as a catch crop on deep water drainage and nitrate leaching in a sweet cherry greenhouse soil. A three-year (2019–2021) field experiment was conducted during the rainfall season from July to September in the post-harvest of sweet cherry, when the plastic cover was removed each year. In the experiment, the five treatments were designed. The three nitrate residue levels denoted by CKR, N1R, and N2R, represented nitrate residue amounts in the soil profile of three nitrogen fertilizer levels(0, 280 and 420kg ha^-1^) before the harvest of sweet cherry(March to June). Two other treatments with and without sweet corn as a catch crop based on the treatments of N1R and N2R were denoted by N1RC and N2RC, respectively. The data of both the spatial and temporal distribution of water and nitrate content during the rainy seasons of 2019, 2020 and 2021 in the field experiment were collected to calibrate and validate the model.

**Results:**

The simulated results have showed that using sweet corn as a catch crop increased the evapotranspiration rate, the upward flux of water and nitrate at a 100 cm soil depth reached a maximum of 1.5 mm d^-1^ and 1.0 kg N ha^-1^d^-1^, respectively, and the downward movement of water and nitrate leached to deeper soil layers was reduced. Compared with CKR, the treatments with catch crops (N1RC and N2RC) reduced the amount of water drainage by 16.4% -47.7% in the 0-180cm soil profile. The average amounts of nitrate leaching in the 1.8 m soil profile during the three-year experiment were 88.1, 113.3, and 58.2 kg N ha^−1^ for the treatment without catch crop (N1R and N2R) and 32.3, 54.8, and 31.4 kg N ha^−1^ for the treatment with catch crop (N1RC and N2RC), respectively. The treatments (N1RC and N2RC) with catch crops decreased the amount of nitrate leaching by 29.6%-69.1% compared with the treatments without catch crops (N1R and N2R).

**Discussion:**

Sweet corn as summer catch crop can reduce nitrate leaching in the sweet cherry greenhouses. Our study has provided an effective method to reduce the risk of nitrate leaching for sweet cherry greenhouses in the North China Plain.

## 1 Introduction

The rapid development of sweet cherry cultivation in greenhouses in the North China Plain (NCP) has attracted considerable attention in the last two decades owing to its high economic benefit (National Bureau of Statistics of China, 2023). However, most fruit greenhouses are mainly run by local orchardists with no or insufficient scientific fertilization knowledge and awareness of environmental protections (Hu et al., 2017; Liu et al., 2019a). Most local orchardists mainly focus on economic benefits and still apply traditional methods to achieve higher fruit yield and quality by applying more synthetic fertilizers (Wang et al., 2022; Zhao et al., 2022). Based on the survey of Shandong, Shanxi, and Liaoning provinces in China, the average nitrogen use efficiency is less than 35% under fruits management and therefor there is a large amount of residual mineral N accumulated in the soil (Han et al., 2015; Lu et al., 2022; Zhu et al., 2022). Liang et al. (2022) reported that the average residual nitrate content in 0–90 cm soil profiles after harvest was 445 kg/ha in the pear orchards in Beijing suburbs. A high content of residual nitrate was not adsorbed by soil because it was negatively charged, soil nitrate is mainly transported through water movement in the soil, and it can easily leach into deep soil horizons when flood irrigation and heavy rains occur (Wang et al., 2010; Zhang et al., 2019; Ahmed et al., 2022).

Because of the high temperatures from June to September, the plastic film covers of most of the sweet cherry greenhouses are removed during the postharvest period in North China. However, more than 50% of the annual rainfall occurs during this period. Abundant rainfall increases the risk of nitrate loss through leaching and causes additional environmental pollution in greenhouse land (Chang et al., 2019; Yu et al., 2019; Bai et al., 2020). Previous studies have shown that summer catch crop planting is an effective method of reducing nitrate leaching in intensively managed greenhouse systems in China (Zhang et al., 2019). Some plants with deep root systems have a high tolerance to high temperature and humidity and the ability to uptake nutrients (Iris et al., 2022, Iris et al., 2023). Meanwhile, accumulated biomass, such as sweet corn, has been widely used for summer catch crops (Guo et al., 2018). Due to the variations in evapotranspiration in the soil without catch crop, planting catch crops will have different degrees of impact on the movement of soil–water at the root and sub-root zone under greenhouse environment conditions, resulting in more complex patterns in soil–water movement, nitrate residual, and leaching (Zhang et al., 2019). However, no specific research has been reported to determine these effects on a quantitative basis in the fruit greenhouse soil.

Direct measurements of water and nitrogen migration in the orchard are expensive and labor-intensive (Bar-Yosef and Sheikholslami, 1976). Alternatively, there have been a number of studies to quantitatively evaluate water drainage and nitrate leaching at the field scale using the simulation models in HYDRUS-1D. These studies mainly considered the effect of irrigation and nitrogen management on nitrate residual and leaching (Vedran et al., 2023; Murphy et al., 2024). This study aimed to use the HYDRUS-1D software package to model dynamics and characteristics of water drainage and nitrate leaching for intercropping catch crop in the sweet cherry greenhouse land with consideration of rainfall timing and intensity scenarios. In this study, three years of field experiment data were collected to evaluate the effects of intercropping catch crop on soil–water storage and nitrogen balance. The data were used to calibrate and validate HYDRUS-1D for simulating and predicting (1) the characteristic differences of soil–water drainage and nitrate leaching between with and without intercropping catch crop and (2) the amount of soil–water drainage and nitrate leaching reductions due to intercropping catch crop under different rainfall conditions.

## 2 Materials and methods

### 2.1 Site details

The experiment was conducted in Changyi, Shandong province, China (42m elevation, 36.91°N and 119.30°E), which is located in a semi-humid temperate region with a mild climate and four distinct seasons. The annual average temperature ranges from 9 to 12°C, with a monthly maximum mean of 30°C in July and a monthly minimum mean of -10°C in January. The frost-free period includes 170–190 d from the end of April to the end of October. Average annual rainfall is 510–550 mm, with approximately 50% falling from June to September. The soil is classified as a Cambisol (FAO/Unesco, 1988). The soil chemical characteristics at a depth of 0–30 cm at the start of the experiment were 53.3 g kg^-1^ organic matter, 3.58 g kg^-1^ total N, 191.2 mg kg^-1^ available P, 589.5 mg kg^-1^ exchangeable K, 7.69 pH, and 1.62 ms cm^-1^ electrical conductivity. Soil physical and hydraulic properties at a depth of 0-180cm soil profile are shown in **Table 1**.

**Table 1:** Physical and hydraulic properties for the soil profile in the experimental site.

Soil layer(cm)	BD (g cm^-3^)	Particle Fraction (%)			Texture (USDA)	*θ_r_* (cm^3^ cm^-3^)	*θ_s_* (cm^3^ cm^-3^)	*α*(cm^-1^)	*n*	*K_s_* (cm d^-1^)
		Sand	Silt	Clay						
0–20	1.38	40.1	46.4	13.5	Loam	0.0473	0.3994	0.0141	1.5776	23.28
20–60	1.56	45.6	43.3	11.1	Loam	0.0445	0.3839	0.0151	1.5323	18.12
60–80	1.59	36.4	52.1	11.5	Silt loam	0.0386	0.3993	0.0153	1.4446	14.38
80–100	1.54	44.9	42.6	12.5	Silt loam	0.0423	0.3862	0.0125	1.5273	27.45
100–150	1.63	39.7	51.2	9.1	Loam	0.0476	0.3853	0.0105	1.4633	16.52
150–180	1.64	45.2	42.4	12.4	Loam	0.0485	0.3816	0.0122	1.5714	14.71

Bd is bulk density; *θ_r_* is the residual water content; *θ_s_* is the saturated water content; *α* is the inverse of the air-entry value; *n* is a pore size distribution index; *K_s_* is the saturated hydraulic conductivity; the pore-connectivity parameter *I* in the hydraulic conductivity function was 0.5 as an average for many soils.

### 2.2 Study greenhouse

The Multi-Span Tunnel solar greenhouse (without solid walls) structure refers to a steel pipe structure of columns and a large truss depth covered with polyethylene film. In the greenhouse, sweet cherry trees (‘Brooks’/’Gisela 6’) were planted in 2013, and tall spindle axes were used as the training system. The sweet cherry trees were planted using cultivation methods in a south-to-north direction at a spacing of 2.0 m (in a row) ×4.0 m (between rows).

### 2.3 Nutrient inputs of the greenhouse

From 2013 to 2017, sheep manure fertilizer was used as the base fertilizer for all trees at an application rate of 65,000 kg hm^-2^. The sheep manure organic fertilizer (organic matter 450.0 g kg^-1^, nitrogen 17.2 g kg^-1^, phosphorus 19.8 g kg^-1^, potassium 24.2 g kg^-1^) was produced by Neimenggu Qingmu Biotechnology Co., LTD. The manure was buried in ditches, and the traditional ditches along both sides of the trees were 0.3 m wide and 0.2 m deep, the distance between ditches and trees was 0.8m. From 2014 to 2017, compound fertilizer(N: P: K = 2:1:0.5)was initially spread on the ground near the tree at an application rate of 450 kg hm^-2^ each year, and then irrigated using the local traditional flood method. From 2018 to 2021, the urea was spread in the traditional ditches in early April, mid-April, early May, and middle May. The amounts of urea applied were 0, 280, and 420 kg N ha^-1^ in different rows of trees. The soil was plowed in middle October. Afterward, sheep manure, phosphorus (p), and potassium (K) fertilizers were spread in the traditional ditches as the base fertilizer for all trees at an application rate of 60,000 kg ha^-1^, 90kg P ha^-1^, and 120 kg K ha^-1^, respectively. Irrigation was conducted at key stages of sweet cherry growth to replenish water. The timing and amount of flood irrigation between the tree rows followed the local methods.

### 2.4 Experimental design

**Figure 1:**
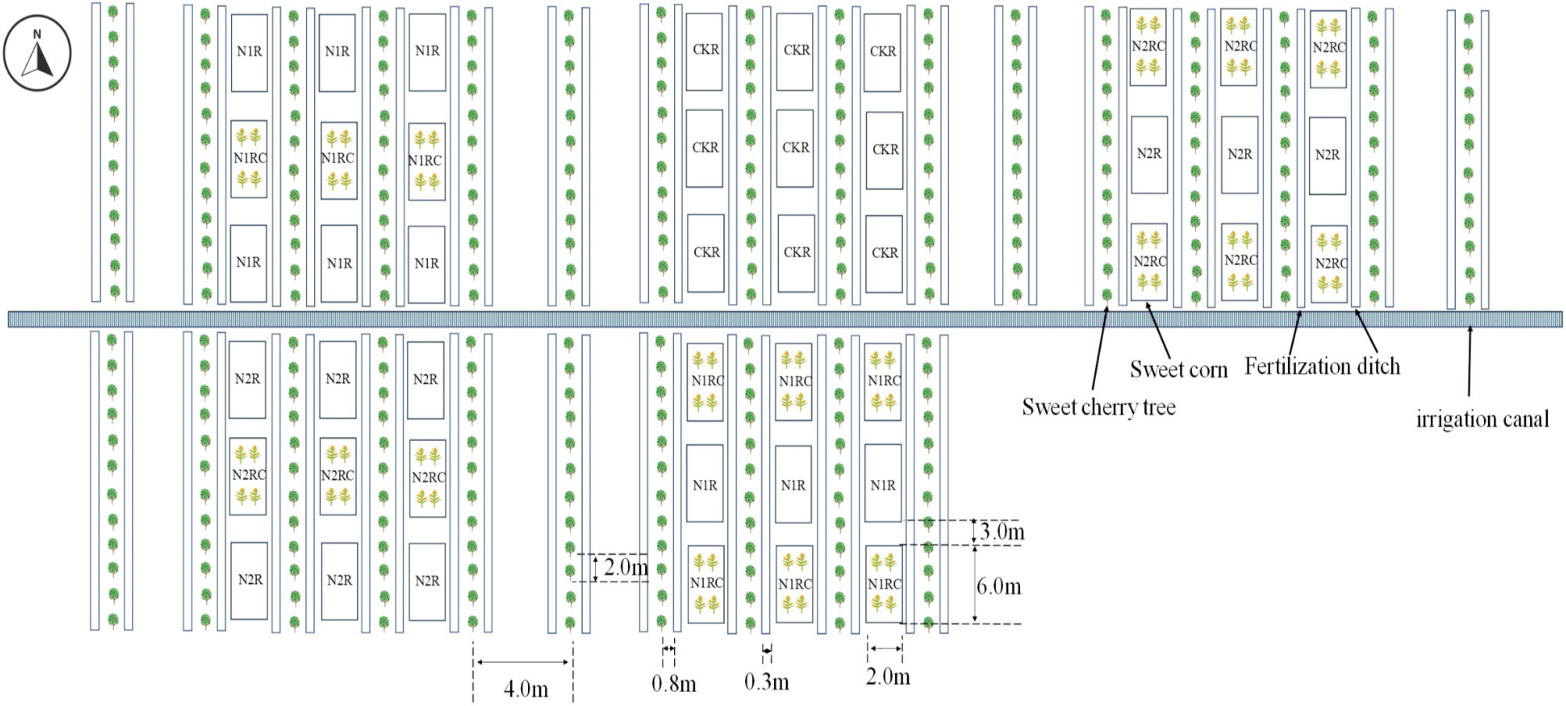
Field layout of the experiment.

**Figure 2:**
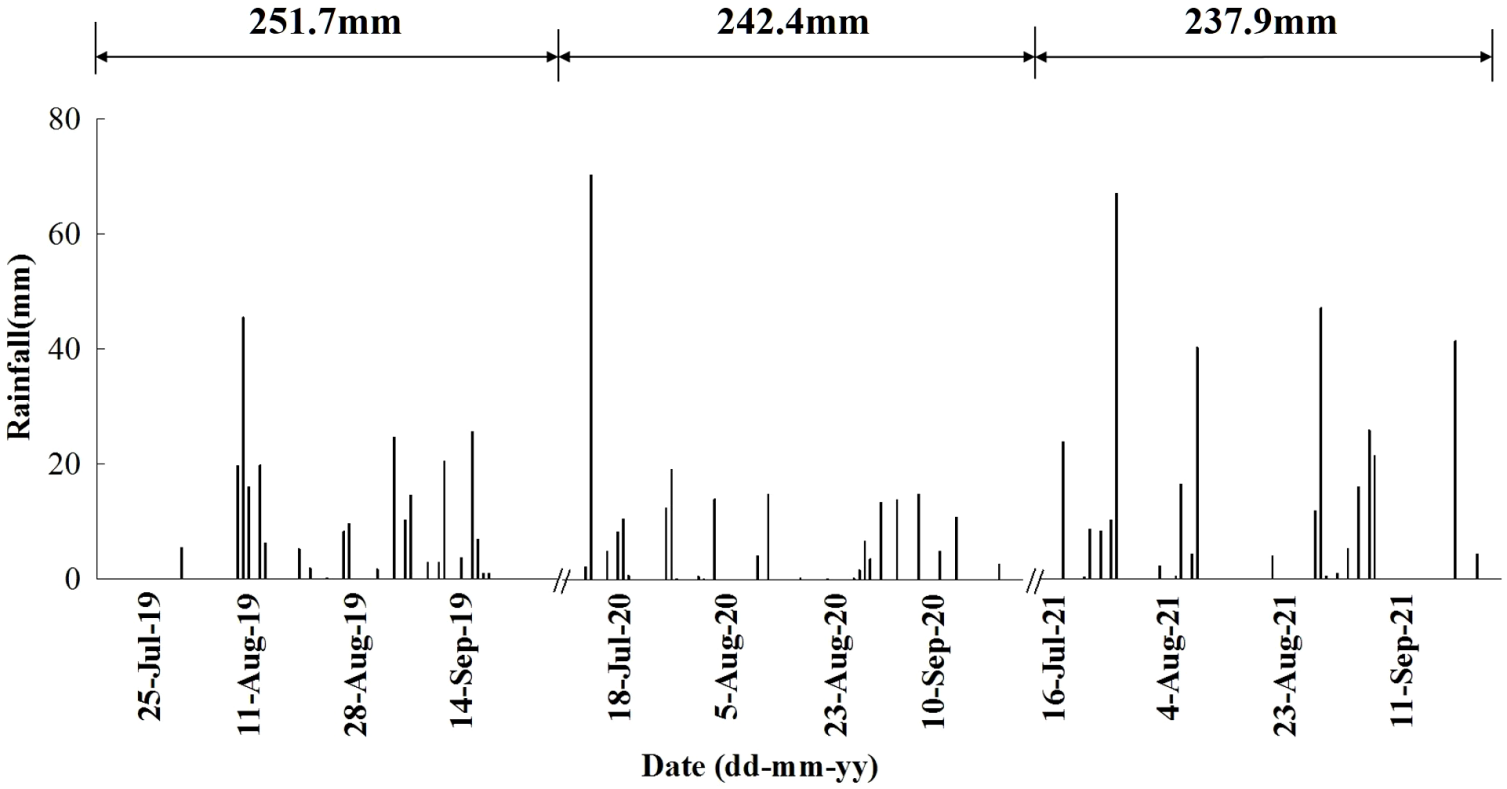
Rainfall during the uncovered summer months of 2019, 2020, and 2021 at the experiment site.

Due to the application of three nitrogen fertilizer amounts (CK:0 kg ha^-1^, N1: 280 kg ha^-1^and N2:420 kg ha^-1^) in the preharvest, there were three nitrogen residue amounts in the soil profile between the tree rows in the postharvest, denoted by CKR, N1R, and N2R. The experiment was conducted in the postharvest period from July 19th to September 30th, 2019, July 16th to September 28th, 2020, and July 14th to September 29th, 2021. All the experiment plots were assigned randomly, having a split-plot arrangement with three factors of nitrogen residual amounts (CKR, N1R, N2R) and two planting patterns (with and without intercropping sweet corn as a catch crop based on the treatments of N1R and N2R, denoted by N1RC and N2RC). Nitrogen residual amounts were used as a main plot, and planting patterns were used as a subplot and replicated three times. Each subplot contains three small fields between tree rows. The small field size was 12m^2^ (6.0m✕2.0m), the distance between the small fields boundary in the south-to-north direction was 3.0 m. Soil ridges were built to prevent the rainfall water from flowing between the small fields in the south-to-north direction. In the catch crop planting field, four rows of sweet corn were planted, and the distances between plants and rows were 0.3m and 0.5m. Field layout of the experiment is shown in **Figure 1**. Before each experimental period, sweet corn seedlings of the “Jingtian 768” variety were nurtured in a greenhouse and then transplanted into plots at the start of the experiment. During the experiment, the plastic film of the greenhouse was removed as common practice. All plots received natural rainfall and were neither irrigated nor fertilized. The amount of rainfall during the experiment is shown in **Figure 2**. As a source of nitrogen, precipitation was limited (4–6 kg N ha^-1^) during the experimental periods and was therefore not included as a nitrogen input.

### 2.5 Field sampling and laboratory analysis

Soil profile pits were excavated at 180 cm depth, and soil samples at different soil texture layers were collected on June 25th 2018 after sweet cherry was harvested. Basic soil properties, including bulk density, pH, EC, SOM, TN, AP and AK were measured. Bulk density was measured using cutting ring method. Soil pH was determined using a pH meter at a soil/water ratio of 1:2.5 (Thunjai et al., 2001). Soil EC of the filtrate from a 1:5 soil–water mixture was measured using a conductivity meter detector (DDS-307) (Bao, 2000). Soil OM was measured using the dilution heat K_2_Cr_2_O_7_ oxidation volumetric method. Total N was measured by Kjeldahl method. Available P was measured by molybdenum-blue method. To determine soil available K, air-dried soil samples were extracted with 1 mol L^−1^ ammonium acetate (soil: solution ratio of 1:10) for 1 h, and the concentration of K in the extract was determined using flame photometry (Bao, 2000).

During the sweet corn growth period, soil samples were collected every 10 days from the subplots to a depth of 180cm at 30cm depth intervals using a 4cm diameter soil auger. Four soil cores per subplot were collected and the samples in the same soil layers were uniformly mixed together. The combined samples were stored in an ice box. Moisture of soil samples was gravimetrically determined at a drying temperature of 105°C. Each fresh soil sample was extracted with 0.1 mol L^-1^ of CaCl_2_ to determine the concentrations of NH_4_^+^-N and NO_3_^−^-N using a continuous flow analyzer (TRAACS 2000, Bran and Luebbe, Norderstedt, Germany). Leaf area index (LAI) was measured at the 6-leaf (V6), 8-leaf (V8), 12-leaf (V12), tasseling (VT), and physiological maturity (R6) stages. The sweet corn was manually harvested on October 2nd, 2019, September 30th, 2020, and September 30th, 2021. Six random plants per subplot, sampled in the harvest period, were dried at 70°C until constant weight and its N contents in cobs, leaves, and stems were analyzed using the Kjeldahl method.

Rainfall, maximum and minimum temperatures, wind speed, and solar radiation were measured daily at the meteorological station on the experimental site.

### 2.6 Model description

The HYDRUS-1D model can be used to simulate the water movement and nitrogen transport. It can also be used to simulate root water and nitrogen uptake for different crops in one-dimensional variably saturated-unsaturated media. The model is suitable for a variety of constant and variable boundary conditions (Simunek et al., 2013).

### 2.7 Soil–water movement

The one-dimensional movement of water in the soil is described using the Richards’ equation (Simunek et al., 2013).


(1) $$\frac{\partial \theta }{\partial t}=\frac{\partial }{\partial z}\left[K\cdot \left(\frac{\partial h}{\partial z}+1\right)\right]-S\left(z,t\right)$$

where *θ* is the volume of the soil–water (cm^3^ cm^-3^); *h* is the pressure head of the soil (cm); *t* is the infiltration time (d); *z* is the spatial coordinate (cm) (upward, positive); *S* (*z, t*) is the rate of water uptake by the roots (cm^3^ cm^-3^ d^-1^); and *K*(*h*) is the rate of unsaturated hydraulic conductivity (cm d^-1^), which is calculated using the van Genuchten model.

### 2.8 Sweet corn root water uptake

The HYDRUS-1D software uses the Feddes model to calculate the rate of water uptake by the root system, which is written as:


(2) $$S\left(z,t\right)=\alpha \left(h,z\right)\cdot \beta \left(z\right)\cdot T_{p}$$

where *α* (*h, z*) is a function of the water stress response (0 ≤ α ≤1); *β(z)* is a function of the standardized root water uptake distribution; *T_p_* is the potential transpiration rate of the crop (cm d^-1^).

In this study, the maximum concentration of solutes for root water uptake was set to 0.1 mg cm^-3^ (Ramos et al., 2012). Due to water stress, root water uptake reductions *α* (*h, z*) were described using the piecewise linear model proposed by Feddes et al. (1978). In the model, water uptake is assumed to be zero at close to saturation (i.e., wetter than some arbitrary “anaerobiosis” point, *h*_1_). The water uptake is at the potential rate when the pressure head is between *h*_2_ and *h*_3_, drops off linearly when *h* > *h*_2_ or *h* < *h*_3_, and becomes zero when *h* < *h*_4_ or *h* > *h*_1_ (*h*_4_ is the wilting point). The pressure head *h*_3_ was adjusted depending on the transpiration rate between 0.1cm d^-1^ and 0.5 cm d^-1^ (Tafteh and Sepaskhah, 2012). The following parameters of the Feddes et al. (1978) model were used as follows: *h*_1_ = −15 cm, *h*_2_ = −30 cm, *h*_3_ = −325 cm to −600 cm, *h*_4_ = −8000 cm.

Phene and Kristensen found sweet corn roots were mainly distributed in 0–120 cm soil layer, and those in 0–120 cm accounted for 95% of the total (Phene et al., 1991; Kristensen and Thorup-Kristensen, 2005).

To avoid overestimation of the nitrogen absorption and water uptake at the root zone, we set the maximum root depth of sweet corn to 110 cm. The sweet corn was transplanted into the field as greenhouse seedlings. Therefore, the initial root depth for the simulation was set to 8 cm. The harvest time was set equal to the duration of the experiments.

### 2.9 Soil NO_3_^−^-N transport

Field experiment results showed that the NO_3_^–^N content was much higher than the NH_4_^+^-N content in the NCP (Ju et al., 2004; Li et al., 2007; Zhang et al., 2019). Similar to the study of Ma et al. (2007); Jin et al. (2007), and Wang et al. (2010), we assumed that NH_4_^+^-N produced through mineralization of organic N was transformed into NO_3_^–^N through nitrification, and soil organic N was mineralized directly into NO_3_^–^N (Hu et al., 2008; Zhang et al., 2019). Thus, the NH_4_^+^-N movement was ignored in the present study. Ammonia volatilization was negligible because no N fertilizer was applied in this study (Hu et al., 2008; Zhang et al., 2019). One-dimensional transport of NO_3_^–^N is described by the advection-dispersion equation.


(3) $$\frac{\partial \left(\theta \cdot C\right)}{\partial t}=\frac{\partial }{\partial z}\left[\theta \cdot D\frac{\partial C}{\partial z}\right]-\frac{\partial \left(q\cdot C\right)}{\partial z}+S_{c}$$

where *C* is the NO_3_^−^-N concentration in the soil solution (mg L^-1^); *D* is the diffusion–dispersion coefficient of NO_3_^−^-N (cm^2^ d^-1^); *q* is the water flux (cm d^-1^); and *S_c_* is the source/sink term, which generally includes the local passive NO_3_^−^-N uptake, mineralization, immobilization, and biological denitrification processes. In this study, the *S_c_* was calculated as follows:


(4) $$S_{c}=c_{s}\times S\left(z,t\right)-k_{\min}-k_{im}\times C-k_{den}\times C$$

where *c_s_* is the NO_3_^−^-N concentration up taken by plant roots (μg cm^-3^), *S*(*z*,*t*) is the root water uptake(d^-1^), *k_min_* is the parameter for the mineralization rate of soil organic nitrogen (zero-order kinetics) (μg cm^-3^ d^-1^), *k_im_* is the parameter for the biological retention rate of soil N_min_ (first-order kinetics) (d^-1^), and *k_den_* is the parameter for the soil denitrification rate (first-order kinetics) (d^-1^).

### 2.10 Boundary and initial conditions

Under actual field conditions, runoff may occur when the supply of water exceeds the soil infiltration capacity. Because this study mainly focused on simulating nitrate leaching, the process was simplified by assuming that all the water had infiltrated the soil and no runoff occurred. An atmospheric boundary condition that allowed surface ponded water was selected for the top boundary. The boundaries of water and nitrate are shown in Equations 5 and 6, respectively, where *E*(*t*) is the time-related function of the soil–water evaporation or the infiltration rate (cm d^-1^).


(5) $$-K\left(\frac{\partial h}{\partial z}+1\right)=E\left(t\right)\;z=0\;t>0$$

(6) $$-\theta \cdot D\frac{\partial h}{\partial z}+qC=q_{0}C_{0}\left(t\right)\;z=0\;t>0$$

The amount of rainfall and the potential evapotranspiration were needed for the simulation analysis. The Penman–Monteith equation was used to estimate the reference crop evapotranspiration level (*ET_0_*), as recommended by the Food and Agriculture Organization of the United Nations (FAO). Then, the potential crop evapotranspiration was calculated using *ET_p_* = *ET_0_ × K_c_*, where *K_c_* is the crop coefficient (Allen et al., 1998). The *K_c_* for sweet corn at stages of transplanting to jointing, jointing to heading, and heading to the grain-filling stage are 0.60, 0.90, 1.21, and 0.7, respectively (Jin et al., 2007; Hu et al., 2008; Wang et al., 2010).

Potential soil evaporation (*E_p_*) was estimated using the empirical expression from the CERES model (Jones and Kiniry, 1986), where *LAI* was measured during the entire growth stage of sweet corn in every catch crop treatment, as shown in Equation 7. *T_p_* (potential corn transpiration) was calculated using Equations 7, 8. In the HYDRUS model, the water stress coefficient was calculated using the Feddes model based on soil suction, as shown in Equation 2. Even though the CKR, N1R, and N2R treatments were bare soil, the *K_c_* value was set to 0.15 (Ramos et al., 2012), and the *LAI* value was set to 1.0 due to the small amount of weed growth (Ritchie, 1972). The calculation method for *E_a_* and *T_a_* was the same as that used to calculate N1RC and N2RC.


(7) $$E_{P}=ET_{p}\left(1-0.43LAI\right)\;LAI\leq 1.0;E_{P}=\frac{ET_{p}}{1.1}e^{-0.4\times LAI}\;LAI>1.0$$

(8) $$T_{P}=ET_{P}-E_{P}$$

The lower boundary was selected to be the soil cross section at a depth of 180 cm, using free-draining boundaries, as shown in Equations 9, 10. The initial soil NO_3_^−^-N concentration and the soil–water concentration of the soil profile were set to values measured at the beginning of each year of the experiment, as shown in Equations 11, 12.


(9) $$\frac{\partial h}{\partial z}=0\;z=-180cm\;t>0$$

(10) $$\frac{\partial C}{\partial z}=0\;z=-180cm\;t>0$$

(11) $$h=h_{0}\left(z\right)\;-180cm\leq z\leq 0\;t=0$$

(12) $$C=C_{0}\left(z\right)\;-180cm\leq z\leq 0\;t=0$$

### 2.11 Model input parameters

Required inputs for the HYDRUS-1D model include the soil hydraulic and nitrogen transport parameters. Functions of soil–water retention, *θ*(*h*), and hydraulic conductivity, *k*(*h*), are estimated using the Mualem (1976) and Van Genuchten (1980) equations, respectively. The hydraulic parameters, including *θ*_r_, *θ*_s_, *K*_s_, *a*, and *n*, were estimated using Rosetta software (Schaap et al., 2001) from the soil particle fractions, bulk density, and soil–water retention. Besides, it has measured the soil particle fraction and the soil water retention by sieve pipette method (Qiu et al., 2021) and pressure membrane meter method (Cresswell et al., 2008), respectively. The range of the nitrogen transformation parameters was determined according to the values reported in the literature (Jin et al., 2007; Hu et al., 2008; Wang et al., 2010). We adjusted soil–water hydraulic parameters and soil solute transport parameters by comparing the simulated and measured datasets. The calibrated soil–water hydraulic parameters and solute transport parameters are listed in **Tables 1**, **2**, respectively.

**Table 2:** Nitrogen transformation and transport parameters for a soil profile of 0–180cm.

Soil layer (cm)	*D*_L_ (cm)	*D*_0_ (cm^2^ d^-1^)	*k_min_^a^* (μg cm^-3^ d^-1^)	*K_im_^b^* (d^-1^)	*K_den_^b^* (d^-1^)
0–20	5.4	10.4	1.5	0.019	0.003
20–40	5.5	10.4	0.7	0.013	0.002
40–90	5.4	10.4	0.2	0.005	0.003
90–120	5.6	10.4	0	0	0.002
120–160	2.3	10.4	0	0	0.001
160–180	2.4	10.4	0	0	0.001

D_L_, longitudinal dispersivity; *D*_0_, molecular diffusion coefficient in free water;

*k_min_*, mineralization rater constant; *K_im_*, immobilization rate constant;

*K_den_*, denitrification rate constant. ^a^ Zero-order kinetics.^b^ First-order kinetics.

The HYDRUS-1D model uses the Arrhenius equation to apply temperature corrections to the transformation parameters. Based on previous studies (Stenger et al., 1995; Ma and Ren, 2004), the activation energy parameter *E_a_* for specific reactions in the simulation process was calculated to be 57142.1 J mol^-1^ for the mineralization, 68576.3J mol^-1^ for the denitrification, and 41900J mol^-1^ for the characterization of the biological immobilization of nitrogen.

### 2.12 Model performance criteria

In this study, three statistical parameters were selected to evaluate the goodness of fit between the simulated and observed values in the field:

(1) Root mean square error
(13) $$RMSE=\sqrt{\sum_{i=1}^{n}\frac{{\left(P_{i}-O_{i}\right)}^{2}}{n}}$$
(2) Nash-Sutcliffe modeling efficiency
(14) $$NSE=1-\frac{{\sum_{i=1}^{n}\left(O_{I}-P_{I}\right)}^{2}}{\sum_{i=1}^{n}{\left(O_{I}-O\right)}^{2}}$$
(3) Index of agreement (*d*)
(15) $$d=1-\frac{\sum_{i=1}^{n}{\left(O_{i}-P_{i}\right)}^{2}}{\sum_{i=1}^{n}{\left(\left|P_{i}-O\right|+\left|O_{i}-O\right|\right)}^{2}}$$

where *P_i_* and *O_i_* are the predicted and observed values, respectively, *O* is the mean of the observed values, and *n* is the number of data pairs. The *RMSE* has a minimum value of 0, with a better agreement close to 0. Modeling efficiency (*NSE*) ranges from −∞ to 1 (Nash and Sutcliffe, 1970). Index of Agreement (*d*) is a measure of the degree to which the predicted variation precisely estimates the observed variation (Willmott, 1981). When *NSE* = 1 or *d* = 1, it indicates a perfect simulation of results. Van Liew and Garbrecht (2003) and Zhang et al. (2019) had suggested that when *NSE*>0.36 and *d*>0.7,the model performed well.

### 2.13 Data analysis

All statistical analyses were performed with IBM SPSS 22.0 (IBM Corp., Armonk, NY, United States). All data were tested for normality before one-way analysis of variance, and Duncan’s multiple range test was run for mean comparisons at a significance level of p<0.05. The graphical presentation was being made using SigmaPlot v.13.

## 3 Results

### 3.1 Effects of planting sweet corn on soil–water storage and nitrogen balance

**Table 3** shows the results for the changes in water storage in the 0–180 cm soil profile during the whole experiment. Due to the large water consumption during the growth period of sweet corn, the soil–water storages of N1RC and N2RC treatments reduced more than those of CKR, N1R, and N2R treatments. In 2019, the soil–water storages of the CKR, N1R, and N2R treatments increased by 74.9, 72.3, and 64.2 mm, respectively. Similar results were obtained in 2020 and 2021.

**Table 3:** Water storage in the 0-180 cm soil profile under different treatments from 2019 to 2021 (mm).

Treatments	2019			2020			2021		
	*W_ini_*	*W_end_*	*△W*	*W_ini_*	*W_end_*	*△W*	*W_ini_*	*W_end_*	*△W*
CKR	374.2±6.8	449.1±3.0	74.9±6.6	455.8±6.8	491.6±11.3	35.8±7.4	450.9±6.3	470.7±9.5	19.8±8.2
N1R	373.1±5.4	445.4±5.2	72.3±9.5	458.2±8.6	490.4±6.9	32.2±4.4	451.7±7.2	471.0±6.9	19.3±3.2
N2R	376.6±9.2	440.8±5.7	64.2±4.0	461.9±12.5	487.9±8.0	26.0±2.9	454.5±6.1	468.3±7.6	13.8±6.7
N1RC	383.2±6.9	363.7±8.9	-19.5±5.5	486.9±7.6	411.8±11.9	-75.1±6.3	461.8±8.9	347.5±8.5	-114.3±7.7
N2RC	379.5±10.7	361.9±6.8	-17.6±5.9	497.8±4.1	397.6±8.6	-100.2±8.6	459.1±11.2	343.4±10.1	-115.7±8.8

*W_ini_*, soil–water storage before sweet corn transplantation; *W_end_*, soil–water storage after end of sweet corn harvest;

*△W*, soil–water storage change; *△W*= *W_end_* - *W_ini_*.

During the summer rainy seasons, the nitrogen balance of the crop system in soil with a depth of approximately 180 cm during the whole experiment was analyzed (**Table 4**). The results show that intercropping sweet corn can significantly reduce the residual N_min_ in the soil compared with the treatments without sweet corn, thus reducing the loss of nitrogen.

**Table 4:** Nitrogen balance in the 0-180cm soil profile under different treatments from2019 to 2021 (kg N ha^-1^).

Year	Treatments	N_min initial_	N_crop_	N_min end_	N balance
2019	CKR	920.9±18.4	−	732.2±31.0 c	188.7±10.4 a
	N1R	1205.7±43.2	−	979.1±87.8 cd	226.6±56.3 ab
	N2R	1468.1±21.4	−	1170.5±46.6 d	297.6±43.5 b
	N1RC	1150.8±83.1	142.9±9.3	811.0±77.1 c	196.9±51.0 a
	N2RC	1333.7±65.3	153.6±8.9	973.3±66.0 cd	206.8±21.9 a
2020	CKR	829.7±14.2	−	735.9±7.8 b	93.8±8.2 a
	N1R	1099.5±73.2	−	826.3±78.7 c	273.2±26.4 cd
	N2R	1372.0±49.5	−	1053.6±66.4 d	318.4±38.4 d
	N1RC	1091.6±58.9	169.6±8.1	753.5±39.7 b	168.5±30.8 b
	N2RC	1194.8±34.2	176.6±17.3	770.4±48.6 cd	247.8±39.0 c
2021	CKR	767.2±5.9	−	733.6±5.8 b	33.6±9.7 a
	N1R	955.9±29.3	−	764.7±9.4 b	191.2±18.7 c
	N2R	1277.7±25.7	−	1035.8±46.4 c	241.9±32.5 d
	N1RC	972.6±67.2	181.7±10.8	679.6±60.2 a	111.3±4.6 b
	N2RC	1021.4±36.8	189.0±15.0	663.3±11.0 b	169.1±19.9 c

N_min_, mineral nitrogen(the sum of NH_4_^+^+NO_3_^-^); N_min initial_,soil N_min_ before sweet corn transplantation; N_crop_,N uptake by sweet corn aboveground; N_min end_, soil N_min_ after end of sweet corn harvest; N balance = N

_min initial_- N_crop_- N_min end_. Values followed by different letters are significantly different at *P* < 0.05.

However, the amount of water drainage and nitrate leaching at a depth of 180 cm was not measured during the experiment periods. Therefore, the patterns of the upward and downward water movement and the transport of soil nitrate in deep soil could not be determined, and the total amount of water drainage and nitrate leaching during all the experimental periods also could not be quantitatively analyzed.

### 3.2 Simulation analysis of soil–water balance and nitrate leaching characteristics during the three-year experimental period

#### 3.2.1 Model calibration and validation

**Figure 3:**
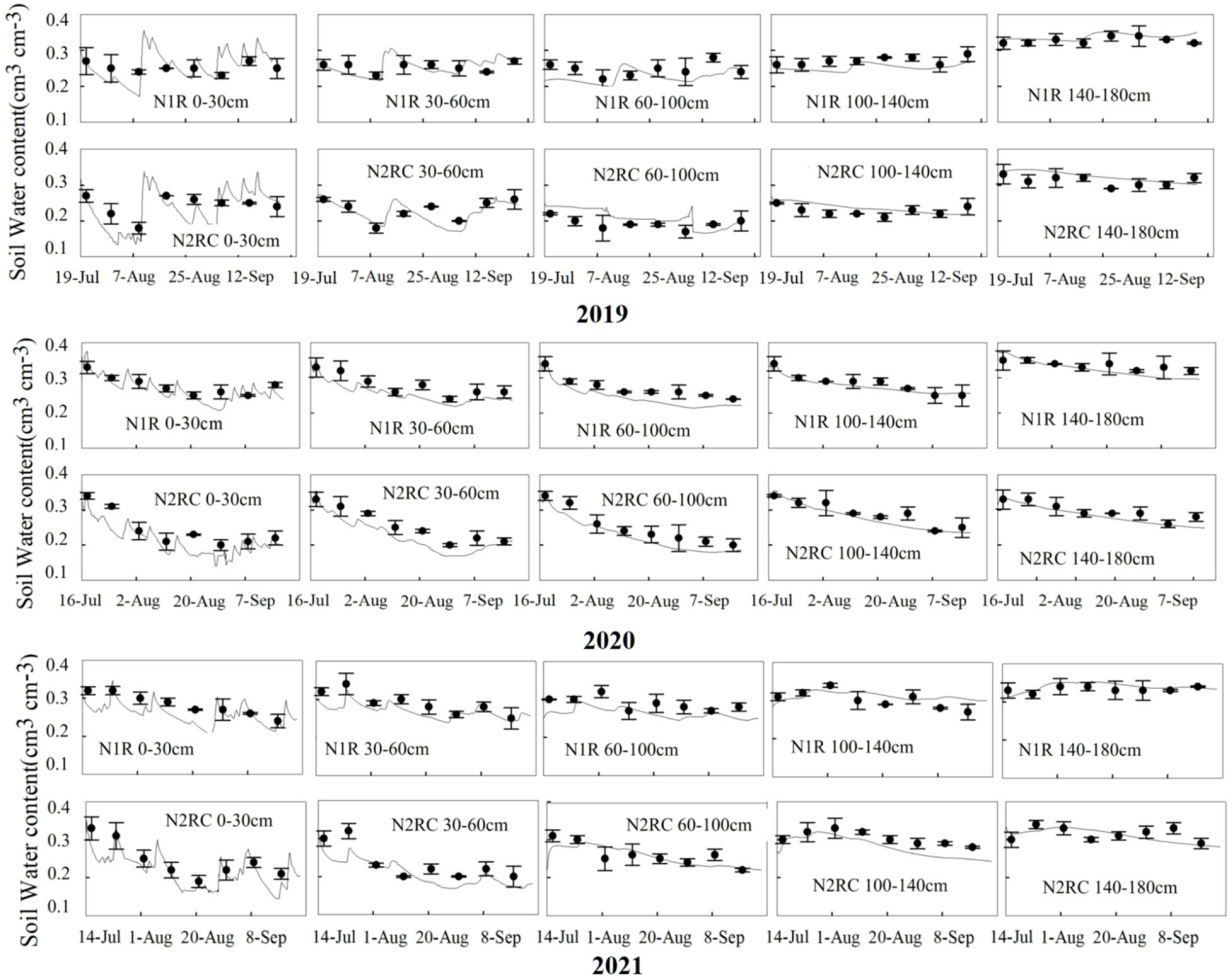
Comparison of simulated (solid lines) and measured (circles dot) volumetric water content (cm3 cm-3) at different depths for N1R and N2RC treatments in 2019, 2020, and 2021 (‘–’ the standard deviation of four replicates).

**Figure 4:**
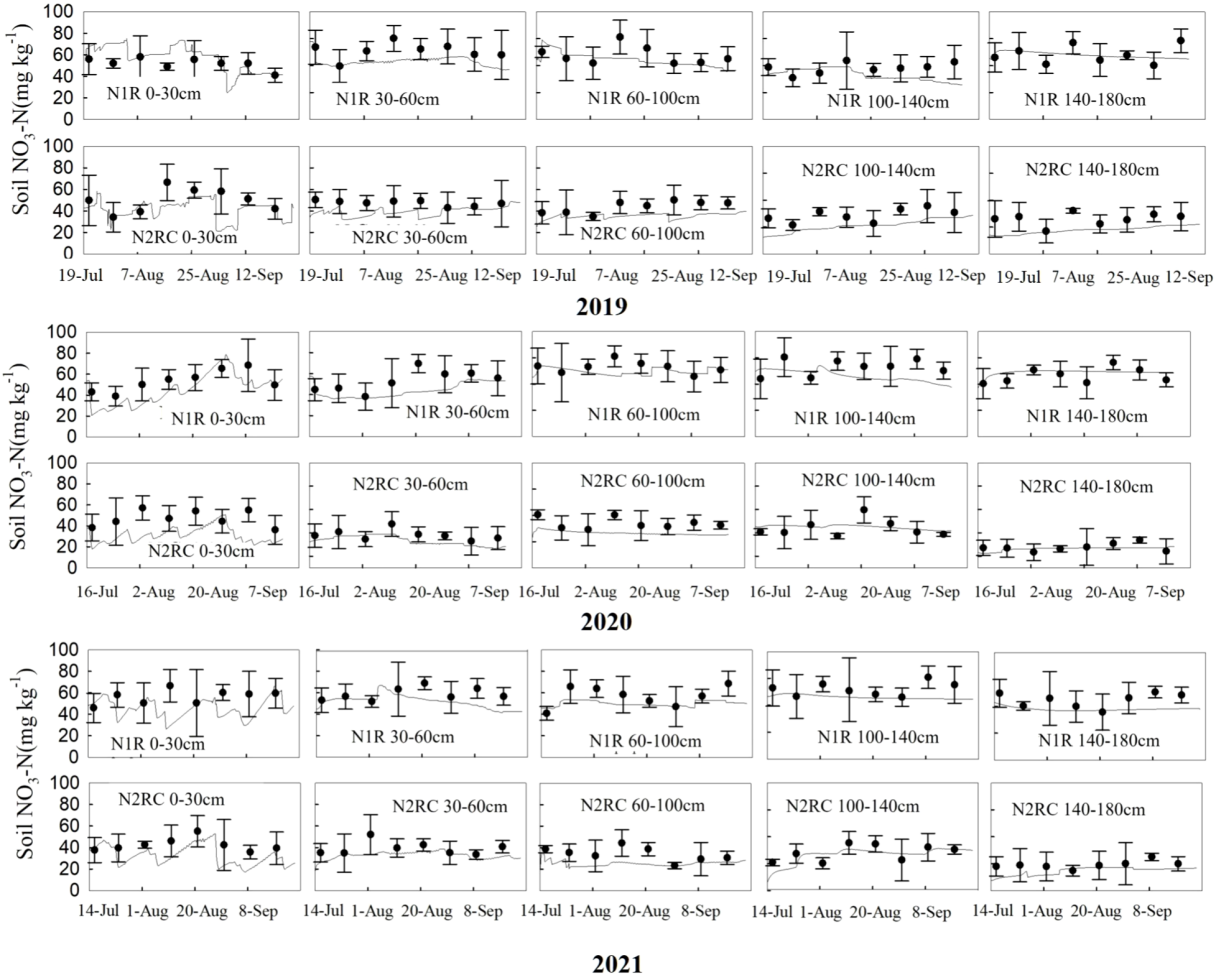
Comparison of measured (circles dot) and simulated (solid lines) soil nitrate N concentration (mg kg-1) at different depths for N1R and N2RC treatments in 2019, 2020, and 2021 (‘–’ the standard deviation of four replicates).

The measured dataset of the 3-year experiments (soil–water content, nitrate concentration, and sweet corn N uptake) from the CKR, N1RC, and N2R treatments were applied to calibrate the model and were used to validate the model from the N1R and N2RC treatments. The model performance statistics *RMSE*, *NSE*, and *d* for water and NO_3_^–^N content at different depths for the calibration and validation dataset are summarized in **Table 5**. For the calibration dataset, *RMSE* values for water content ranged from 0.01 to 0.07 cm^3^ cm^−3^. *NSE* values ranged from -0.22 to 0.85, and *d* values ranged from 0.76 to 0.92. The *RMSE* of NO_3_^–^N concentration values in different soil depths ranged from 7.25to 12.41 mg kg^−1^. *NSE* values ranged from -0.36 to 0.50, and *d* values ranged from 0.59 to 0.79. For the validation dataset, the *NSE* values of validated treatments for soil water content were positive at 30–180 cm soil depths, ranging from 0.35 to 0.87. For soil NO_3_^−^-N concentration, the *NSE* values of validated treatments are positive in 60–180 cm deeper soil layer, ranging from 0.34 to 0.53. In addition, the *d* values for soil water content and NO_3_^−^-N concentration ranged from 0.72 to 0.95 and 0.44 to 0.80 under N1R and N2RC treatments, respectively. The *RMSE* values of the soil–water for validated treatments decreased from 0.06 cm^3^ cm^-3^ in the 0–30 cm soil profile to 0.01 cm^3^ cm^-3^ in the 100–180 cm soil profile, showing good agreement between simulated and measured soil water content (Hu et al., 2010; Sun et al., 2013). The *RMSE* values for nitrate concentrations ranged from 6.61 to 12.95 mg kg^-1^, which was also acceptable (Nangia et al., 2008; Guo et al., 2010; Sun et al., 2013). The results indicate that the model performed reasonably well in simulating soil–water content and nitrate concentration in the experiment area (**Figures 3**, **4**).

**Table 5:** Model performance statistics of predicted soil–water content and NO_3_^−^-N concentration at different depths for the calibration treatment (CKR, N1RC, and N2R) and validation treatments (N1R and N2RC).

Items	Depth (cm)	CKR			N1RC			N2R			N1R			N2RC		
		*RMSE*	*NSE*	*d*	*RMSE*	*NSE*	*d*	*RMSE*	*NSE*	*d*	*RMSE*	*NSE*	*d*	*RMSE*	*NSE*	*d*
Water content	0–30	0.07	-0.20	0.79	0.07	-0.22	0.76	0.06	-0.21	0.77	0.06	-0.25	0.78	0.06	-0.32	0.72
	30–60	0.06	0.34	0.82	0.05	0.33	0.80	0.05	0.32	0.82	0.04	0.35	0.83	0.05	0.38	0.79
	60–100	0.04	0.72	0.81	0.03	0.69	0.85	0.03	0.64	0.85	0.02	0.77	0.92	0.02	0.69	0.86
	100–140	0.04	0.79	0.90	0.01	0.82	0.89	0.01	0.80	0.88	0.01	0.86	0.95	0.02	0.82	0.91
	140–180	0.03	0.84	0.92	0.01	0.85	0.92	0.01	0.84	0.93	0.01	0.87	0.95	0.01	0.85	0.93
NO_3_^-^-N concentration	0–30	11.02	-0.35	0.59	12.41	-0.36	0.61	11.96	-0.32	0.60	12.95	-0.37	0.64	12.47	-0.48	0.44
	30–60	10.04	-0.07	0.62	11.95	-0.09	0.73	11.61	-0.08	0.72	12.06	-0.12	0.77	11.69	-0.29	0.59
	60–100	11.21	0.38	0.71	10.98	0.31	0.74	10.24	0.33	0.73	10.14	0.34	0.79	11.65	0.42	0.68
	100–140	9.56	0.47	0.78	9.64	0.40	0.77	9.25	0.41	0.75	8.69	0.41	0.76	8.32	0.49	0.71
	140–180	7.25	0.45	0.77	7.53	0.43	0.79	7.31	0.50	0.77	6.61	0.47	0.80	7.19	0.53	0.76

**Figure 5:**
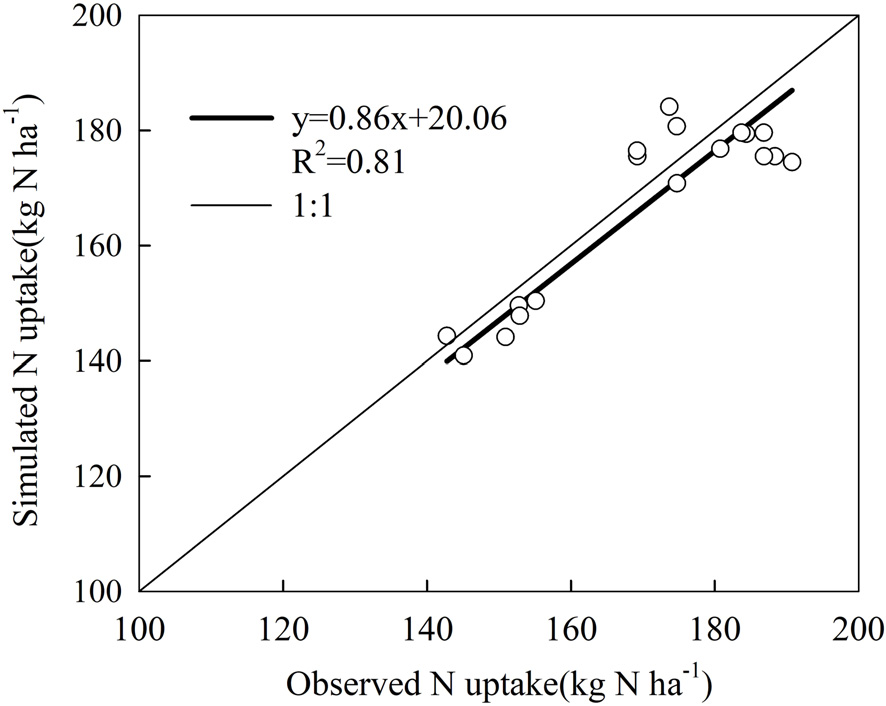
Comparison of simulated and observed sweet corn N uptake of calibration and validation.

A comparison of simulated and measured sweet corn N uptakes is shown in **Figure 5**. The correlation coefficient of crop N uptake was 0.81, which was at a significant level with *P* < 0.001. The simulated results showed that the HYDRUS-1D model performed reasonably well in predicting the water movement, nitrate transport, and sweet corn N uptake in this study.

#### 3.2.2 Dynamic of soil–water drainage and nitrate leaching

**Figure 6:**
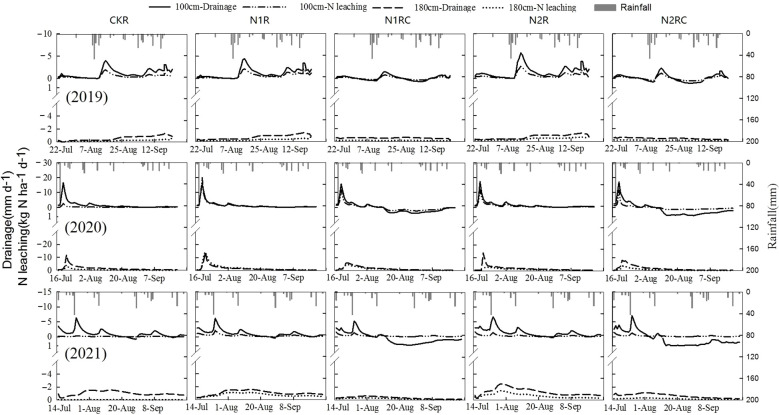
Dynamic water drainage and N leaching below 100 cm and 180 cm soil depths for all treatments.

The daily water drainage and nitrate leaching under five treatments during the three-year experiments are shown in **Figure 6**. A negative value represents water drainage and nitrate leaching below 100 and 180 cm, while a positive value represents the upward flux of water and nitrate in the soil at a depth of 100 cm. Large peak values for water drainage and nitrate leaching were always simulated after a heavy rainfall event. In 2019, 46 mm d^-1^ of rainfall was received on August 11th, which resulted in the largest peak in water drainage and nitrate leaching on August 16th for all treatments. On that day, the maximum daily water drainage rate was 1.6 mm and 4.4 mm for the treatments with and without catch crop, respectively, indicating that water uptake by sweet corn roots reduced the risk of water drainage. Nitrate leaching always occurs after a large amount of water drainage event. In addition, planting sweet corn can reduce the rate of nitrate leaching. For example, the largest daily amount of nitrate leaching after the rainfall process on August 17th under catch crop treatments at a 100 cm soil depth was 0.8 kg N ha^-1^, which was 150% lower than treatments without catch crop.

For the seasons of 2020 and 2021, heavy rainfall occurred at an early stage of sweet corn development. Therefore, the catch crop treatments did not effectively prevent water drainage and nitrate from leaching due to the shallow root system. For example, on July 18th, the largest daily amounts of nitrate leaching were 19.2 and 15.8 kg N ha^-1^, accounting for 75% and 82% of seasonal nitrate leaching for the treatments with and without sweet corn, respectively. Similar results were simulated on July 22nd, 2021.

Compared with the peak values at a soil depth of 100 cm, the water drainage and nitrate leaching curves at a 180 cm soil depth were relatively flat and had daily values of less than 3 mm and 2 kg N ha^-1^, respectively, with the exception of the largest peak values on July 18th, 2020.

During the three-year experiments, sweet corn generated an upward flux of water at a 100 cm soil depth from the middle to the end of the experiment, which could reduce the risk of nitrate leaching to some extent. However, the upward daily amount of water and nitrate were simulated to be relatively low and never exceeded 1.5 mm and 1.0 kg N ha^-1^, respectively.

#### 3.2.3 Soil–water balance

The results for the water balance in the 0–100 and 0–180 cm soil profiles during the whole experiment are shown in **Table 6**. The main water consumption was evapotranspiration, and the mean values of the catch crop treatments for the three-year experiment were 224.4, 258.9, and 304.4 mm, accounting for approximately 89.6%, 106.8% and 127.9% of the total water input (rainfall), respectively. These values are higher than those of the treatments without sweet corn. As a result, the amount of water drainage at a 100 cm soil depth under catch crop treatments was significantly lower than that under the treatments without sweet corn despite some exceptions. Similar results were simulated at a 180 cm soil depth due to two reasons. First, the lower drainage at 100 cm soil depth might reduce the downward flux of water and significantly reduce water drainage at deeper soil. Second, the water drainage mainly occurred from the 0–100 cm range within the 0–180 cm soil depth. Planting sweet corn did not cause fundamental changes to the overall trend of soil–water movement below the root zone, but it might have reduced the downward flux of water and reduced the amount of water drainage (**Figure 6**).

**Table 6:** Water balance in the 100 cm and 180 cm soil profiles under different treatments.

Year	Treatments	*R*(mm)	*E*(mm)	*T*(mm)	*D*_100_(mm)	*D*_100_ */R*	*△W*_100_(mm)	*D*_180_(mm)	*D*_180_ */R*	*△W*_180_(mm)
2019	CKR	251.7	116.4±1.7	−	71.1±2.5 c	28.2±1.4% c	64.2±2.7	59.7±3.1 c	23.7±1.2% c	75.6±4.5
	N1R		117.9±1.4	−	74.8±3.7 c	29.7±2.1% c	59.0±4.5	62.9±4.8 c	24.9±2.4% c	70.9±6.2
	N2R		116.1±1.5	−	84.9±2.6 d	33.7±1.1% d	50.7±3.7	69.4±2.2 d	27.6±1.1% d	66.2±2.8
	N1RC		50.5±1.1	177.8±2.1	28.1±3.9 a	11.2±1.6% a	-4.7±1.1	38.7±2.6 a	15.4±1.3% a	-15.3±2.4
	N2RC		44.1±0.7	176.4±2.2	40.2±2.2 b	15.9±0.8% b	-9.0±2.2	45.0±4.0 b	17.8±2.0% b	-13.8±5.4
2020	CKR	242.4	125.4±1.2	−	97.2±4.9 b	40.1±2.1% b	19.8±5.6	113.8±3.69 b	46.9±2.0% b	3.2±0.5
	N1R		126.7±1.0	−	99.4±3.8 b	41.0±1.6% b	16.3±3.2	111.8±3.56 b	46.1±1.9% b	3.9±0.8
	N2R		125.9±1.3	−	96.3±3.4 b	39.7±1.8% b	20.2±2.6	121.5±7.7 b	50.1±1.9% b	-5±1.2
	N1RC		56.5±1.2	192.4±0.7	59.0±5.8 a	24.3±2.5% a	-65.5±4.3	92.8±5.9 a	38.3±2.0% a	-99.3±5.8
	N2RC		66.2±0.9	202.8±2.3	51.1±3.5 a	21.1±1.5% a	-77.7±2.0	97.5±7.3 a	40.2±3.9% a	-124.1±9.5
2021	CKR	237.9	165.0±0.9	−	74.6±4.0 b	31.4±2.2% b	-1.7±0.2	79.7±4.4 b	33.5±2.4% b	-6.8±1.0
	N1R		164.7±0.6	−	81.7±5.0 b	34.3±2.8% b	-8.5±2.5	75.8±4.2 b	31.8±2.3% b	-2.6±0.4
	N2R		165.1±0.9	−	93.5±4.4 c	49.3±1.9% c	-20.7±5.3	110.4±9.3 c	46.4±5.1% c	-37.6±8.3
	N1RC		65.4±1.1	241.8±6.9	30.3±7.3 a	12.7±3.2% a	-99.6±8.9	39.8±3.5 a	16.7±1.9% a	-109.1±4.6
	N2RC		62.9±1.6	238.7±3.8	24.9±3.5 a	10.4±1.5% a	-88.6±6.8	43.5±3.5 a	18.3±1.9% a	-107.2±7.3

*R*, rainfall(mm); *E*, soil evaporation(mm); *T*, crop transpiration; *D*_100_, water drainage below 100cm soil profile; *D*_180_, water drainage below 180cm soil profile;*△W*, soil–water storage change*, △W=R-E-T-D.* Values followed by different letters are significantly different at *P* < 0.05.

The soil–water storage of the catch crop treatments for the three-year experiment in a 100 cm depth soil profile negatively changed, indicating that the crop requirement exceeded the rainfall amount. For the treatments without sweet corn, the soil–water storage positively changed. Similar results were obtained for the soil profile with a 180 cm depth.

#### 3.2.4 Nitrate leaching

Nitrate leaching in the 0–100 cm and 0–180 cm soil profiles is shown in **Table 7**. In 2019, during the experiment, the amount of the initial accumulated nitrate in the 100 cm soil profile under the N2R treatment was the highest, reaching 1153.9 kg ha^-1^, the amount and the percentage of N leaching were 93.5 kg ha^-1^ and 8.1%, which was significantly higher than that of the CKR and N1R treatment, respectively. Therefore, a larger amount of the initial accumulated nitrate in the soil profile results in a higher leaching percentage. Under conditions of a similar initial accumulated amount of nitrate in the 0-100 cm soil profile, the accumulative amount of nitrate leaching for the N2RC and N1RC treatments decreased to 24.5kg ha^-1^ and 20.9 kg ha^-1^, the percentage of N leaching also decreased to 2.3% and 2.5%, respectively, which was significantly lower than that of the N2R and N1R treatments. Similar results were obtained in 2020 and 2021. Under the N2RC and N1RC treatments, the amount of nitrate leaching ranged from 26.7 to 34.1kg ha^-1^ and 18.4 to 22.1 kg ha^-1^, accounting for approximately 2.8–4.0% and 2.7-2.9% of the initial accumulative amount of soil nitrate, which remained significantly lower than that of the N2R and N1R treatment, respectively. In the 0-180 cm soil profile, the amount and the percentage of N leaching under catch crop treatments was also significantly lower than those under the treatments without catch crop during the three-year experiment. The results showed that planting sweet corn decelerated the nitrate leaching down the soil profile.

**Table 7:** Nitrate leaching in the soil profile under different treatments (kg N ha^-1^).

Year	Treatments	0–100 cm soil profile			0–180 cm soil profile		
		N_ini_	N_lea_	N_lea_/N_ini_	N_ini_	N_lea_	N_lea_/N_ini_
2019	CKR	620.4±44.0	33.0±2.0	5.3±0.2% c	837.2±27.3	55.5±4.4	6.6±0.5%d
	N1R	891.7±63.1	53.8±4.2	6.0±0.2% c	1131.0±57.4	68.2±5.5	5.1±0.2% c
	N2R	1153.9±80.9	93.5±2.1	8.1±0.4% d	1378.8±21.9	107.9±1.6	7.3±0.1% d
	N1RC	834.7±56.9	20.9±1.5	2.5±0.2% a	1100.2±78.6	31.1±0.7	2.8±0.2% a
	N2RC	1055.2±48.9	24.5±1.9	2.3±0.2% a	1237.4±67.5	33.4±1.1	2.7±0.2% a
2020	CKR	446.0±13.9	24.9±0.4	5.6±0.8% c	766.1±15.7	67.4±1.0	8.8±0.7% c
	N1R	797.7±92.1	69.6±1.5	8.8±0.8% d	1019.5±61.7	71.5±4.6	7.0±0.4% b
	N2R	902.2±97.9	95.2±7.7	10.6±0.4% e	1298.6±42.8	155.1±16.7	11.9±0.8% d
	N1RC	752.5±23.1	22.1±0.3	2.9.±0.1% a	1024.3±50.8	50.3±0.3	4.9±0.2% a
	N2RC	864.6±64.0	34.1±2.4	4.0±0.5% b	1122.5±30.4	58.9±1.3	5.2±0.4% a
2021	CKR	414.2±11.7	19.1±0.2	4.6±0.5% c	685.5±7.4	31.65±0.3	4.6±0.2% b
	N1R	807.1±22.1	26.5±0.9	3.3±0.1% b	908.1±32.5	49.0±1.3	5.4±0.1% bc
	N2R	859.2±28.1	31.0±2.3	3.6±0.2% b	1194.4±23.3	67.3±0.4	5.6±0.4% bc
	N1RC	687.7±37.7	18.4±0.7	2.7±0.4% a	881.6±60.8	28.2±2.5	3.2±0.2% a
	N2RC	791.0±9.7	26.7±1.5	2.8±0.2% a	952.7±17.2	34.7±1.9	3.6±0.2% a

N_ini_, initial nitrate; N_lea_, Nitrate leaching.

Values followed by different letters are significantly different at *P* < 0.05.

#### 3.2.5 Scenario simulation analysis on prevention of soil nitrate leaching with catch crop under heavy rainfall conditions

**Figure 7:**
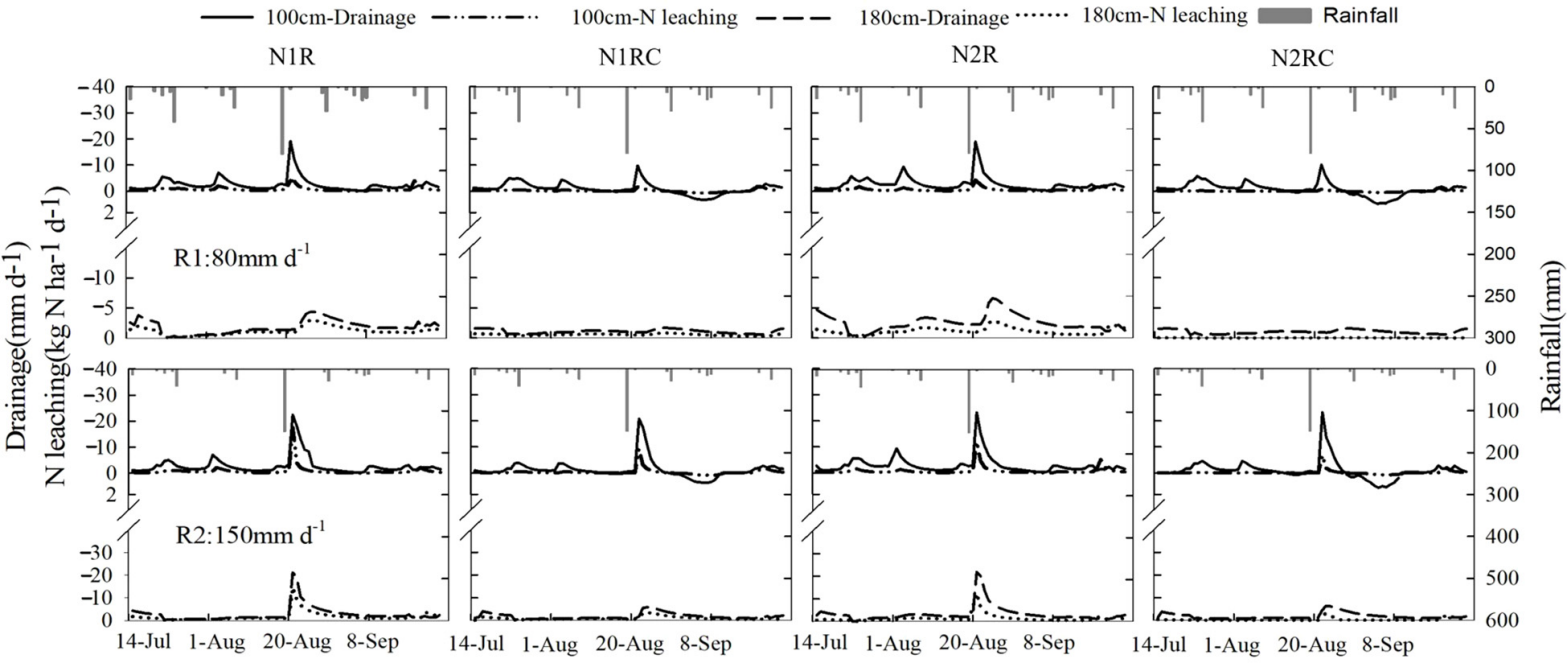
Dynamics of water drainage and N leaching below 100 cm and 180cm soil depths for all treatments under heavy rainfall scenarios.

After examining historical meteorological data (1981–2018), we found that although the total rainfall from 2019 to 2021 was similar to the historical average value (238 mm) for the same period, the highest daily maximum rainfall (68 mm) during 2019 to 2021 was less than the historical daily maximum (80–157 mm). In order to simulate the effects of heavy rainfall in the summer season on soil–water drainage and nitrate leaching in soil with and without sweet corn, two scenarios were simulated by changing the top boundary conditions in the model (daily rainfall R1:80 mm and R2:150 mm) using the test data collected in 2021. The total rainfall in the simulation consisted of natural rainfall and heavy rainfall. Simulated heavy rainfall was set to occur on August 22nd. As shown in **Figure 7**, the dynamics of soil–water drainage and nitrate leaching indicate that the heavy rainfall resulted in a large amount of water drainage at a 100 cm soil depth for all treatments. In addition, the peaks of daily nitrate leaching under the N1R and N2R treatments increased from 9.5 and 8.6 kg N ha^-1^ to 17.5 and 11.9 kg N ha^-1^, respectively, indicating that a larger amount of the initially accumulated nitrate in soil profile caused a higher percentage of nitrate leaching to deeper depth when the rainfall intensity increased. Compared with the treatments without sweet corn, intercropping sweet corn decreased the amount of daily nitrate leaching peaks by 46.1% in the R1 scenario. However, in the R2 scenario, water drainage of the catch crop treatments was similar to that of the treatments without sweet corn, which increased the peaks of maximum daily nitrate leaching to 9.6 kg N ha^-1^. Under the R2 scenario, the catch crop treatments reduced the amount of nitrate leaching by 28.9% at a 180 cm soil depth compared with those at the 100 cm depth. This result showed that as daily rainfall intensity increased, the ability of sweet corn to restrict nitrate leaching at the sub-root zone also decreased.

## 4 Discussion

During the years of fruit production in the greenhouse, farmers usually engage in high N fertilization practices for the management of sweet cherries to ensure high yields of the marketable products, which leads to excessive accumulation of mineral N in the soil profile. Our study confirmed that mineral N in the sweet cherry greenhouse soil profile was mainly in the form of nitrate, which is consistent with the results in Northern and Central China (Ju et al., 2004; Guo et al., 2010). The average residual nitrate amounts of the N1R and N2R treatments in the 0-180 cm soil profile after sweet cherry was harvested in 2019, 2020, and 2021 were 1254.9, 1159.0, and 1051.3 kg N ha^-1^, respectively. These results are similar to the results in some studies in Northern China, where the residual NO_3_^–^N in 2 m soil profiles was greater than 1000 kg ha^-1^ in the greenhouse orchard (Gao et al., 2021; Zhu et al., 2023). During the summer rainy period, when sunlight greenhouses have open roofs, residual nitrate leaching causes severe environmental problems, such as groundwater nitrate pollution. The rational application of nitrogen fertilizer is undoubtedly a fundamental requirement to control the amount of nitrate leaching (Zhao et al., 2022; Ahmed et al., 2022), while another efficient method is planting catch crops in the period when the plastic film covering the sunlight greenhouses was removed (Fan et al., 2021). In this study, under conditions of similar initial accumulated amounts of NO_3_^–^N in the soil profile, the amount of nitrate leaching in the treatments without sweet corn was 16.1–281.6% higher than that in the catch crop treatment in the 0-100 cm soil profile. Catch crops reduced the potential for NO_3_^–^N leaching by taking up soil active N during soil–water recharge, absorbing and transpiring water, and reducing water drainage (Li et al., 2021; Diana et al., 2022). In this study, the amount of N taken up by sweet corn was 142.9–189.0 kg ha^-1^, which was comparable to the value of 154–187 kg N ha^-1^ in Beijing, as reported by Guo et al. (2018). Compared with the treatments without sweet corn, sweet corn cropping reduced N leaching by 21.2–96.2 kg N ha^-1^ in the 180 cm deep soil zone was achieved, which was lower than the results reported by Guo et al. (2008) and Zhang et al. (2019) in Northern China. This phenomenon could be attributed to soil organic N mineralization, which was not considered an N input factor in our study and might partly be responsible for the differences above.

Because the transpiration intensity of sweet corn changes with meteorological factors, this raises the question of whether the amount of nitrate leaching was much different during the three-year experiments. However, one of the limitations of this study was that the depth of the sweet corn root was set at 110 cm for all simulations, which could affect the amount of water drainage and nitrate leaching in different years. The amounts of nitrate leaching under catch crop treatment in the 0–100cm soil profile in 2019, 2020 and 2021 were 20.9–24.5, 22.1–34.1 and 18.4–26.7 kg N ha^-1^, with the leaching percentages of 2.3-2.5%, 2.9-4.0% and 2.7-2.8%, respectively. The results show that due to the occurrence of fewer extreme climatic events, such as heavy rainfall and drought, during the experiment period, the amount of nitrate leaching in the 0–100 cm soil profile under catch crop treatment had no large differences under similar meteorological conditions. The amount of nitrate leaching depends on the integrated influences of rainfall (or irrigation) and nitrate concentration in the soil profile (Tafteh and Sepaskhah, 2012; Su et al., 2022). The larger the amount of the initial accumulated NO^3–^N and water drainage in the soil profile, the higher the percentage of N leaching (Zhu et al., 2022; Wang et al., 2024). In contrast, the amount of nitrate leaching will be decreased if one factor is restricted (Liu et al., 2019b). Though the amounts of evapotranspiration under sweet corn treatment in 2020 and 2021 were higher than that in 2019 (i.e., with increases in 34.6 mm and 80.0mm, respectively), the differences in water drainage and nitrate leaching amount out of the 100cm soil profile were insignificant. Similar results were presented by Wang et al. (2014) and Chilundo et al. (2018), who pointed out that water drainage and nitrate leaching mainly occurred during heavy rainfall events and were less affected by the water consumption of maize. The results were in agreement with our heavy rainfall scenario simulations.

Because of the experiment design and data deficiency, the dataset used for the calibration and validation model was obtained under the same meteorological conditions and soil environment, which are not completely independent. However, some studies have also calibrated and validated the model using data obtained from different treatments in the same years and the same soil environment or same treatments in different years (Wang et al., 2010; Hu et al., 2010; Sun et al., 2013). Azad et al. (2023) and Thao et al. (2024) suggested that the dataset for calibration and validation of the water and nitrogen management model should be conducted for different climates and soil environments. Otherwise, it will limit the extrapolation of the performance of the model in other pedoclimatic and field circumstances. Therefore, the experiment design of this study, model calibration, and validation procedures need to be further investigated in various field environments.

## 5 Conclusion

Founded on the data of a three-year experiment in Changyi, Shandong Province, this study has calibrated and validated the HYDRUS-1D model. The results have showed that the simulated soil water content, NO_3_^−^-N concentration and crop uptake of N matched well with the measured data. The HYDRUS-1D model could be a useful tool for simulating and evaluating the effects of catch crops on deep soil water drainage and nitrate leaching in a sweet cherry greenhouse soil during the summer open roof period.

Sweet corns as summer catch crop could absorb the residual N in the soil, decrease the soil water deep drainage and prevent nitrate leaching during rainy summer season. Compared with CKR, intercropping sweet corn significantly decreased the amount of water drainage by 16.4%-47.7% due to evapotranspiration. However, through simulations it displayed that the reduction range lowered with the increase in rainfall intensity. Intercropping sweet corn also helped to reduce the amount of nitrate leaching by 29.6%-69.1% compared with CKR. The nitrate was mainly accumulated in the 0-100cm soil profile after sweet cherry was harvested. Sweet corn with deep root would be more effective in reducing N leaching. It’s concluded that intercropping sweet corn was capable of reducing the risk of nitrate leaching in intensive sweet cherry greenhouse with open roof in the North China Plain.

## Data Availability

The original contributions presented in the study are included in the article/supplementary material. Further inquiries can be directed to the corresponding author.
